# MicroRNAs as potential indicators of the development and progression of uterine leiomyoma

**DOI:** 10.1371/journal.pone.0268793

**Published:** 2022-05-31

**Authors:** Myungshin Kim, Dain Kang, Mi Yeon Kwon, Hee Jeong Lee, Min Jeong Kim

**Affiliations:** 1 Department of Laboratory Medicine, College of Medicine, The Catholic University of Korea, Seoul, Republic of Korea; 2 Catholic Genetic Laboratory Center, Seoul St. Mary’s Hospital, College of Medicine, The Catholic University of Korea, Seoul, Republic of Korea; 3 Clinical Medicine Research, Bucheon St. Mary’s hospital, The Catholic University of Korea, Bucheon, Republic of Korea; 4 Department of Pathology, College of Medicine, The Catholic University of Korea, Seoul, Republic of Korea; 5 Department of Obstetrics & Gynecology, Buheon St. Mary’s Hospital, College of Medicine, The Catholic University of Korea, Seoul, Republic of Korea; Kunming University of Science and Technology, CHINA

## Abstract

Recent studies demonstrated a significant role of several microRNAs (miRs) in the development of leiomyoma. Here, we investigated miR expression profiles using microarray and found a significantly higher expression of miRs in leiomyoma than in adjacent myometrium. We also confirmed the upregulation of five selected miRs including miR-181a-5p, 127-3p, 28-3p, 30b-5p and let-7c-5p in cellular proliferation, extracellular matrix turnover, and angiogenesis by RT-qPCR. Interestingly, the miRs showed a higher expression in cases of large leiomyoma or in patients with a history of transfusion due to anemia. We then analyzed the expression of the miR target molecules including Transforming Growth Factor Beta Receptor 2 (TGFBR2) and Insulin-like Growth Factor 2 mRNA Binding Protein 1 (IGF2BP1) via immunohistochemistry. TGFBR2 and IGF2BP1 were positively stained in 81% and 62.5% of leiomyoma tissues but not in adjacent myometrium. Both were more frequently positive in patients with ≥ 6 cm leiomyoma and mass effect. The mean expression levels of miR-181a-5p, 127-3p, 28-3p, 30b-5p and let-7c-5p were higher in cases with TGFBR2 and IGF2BP1 positive leiomyoma. We observed several miRs were overexpressed in leiomyoma tissues, and these results provide insight into the role of miRs in the development and progression of leiomyoma and underscore the need to validate their utility as diagnostic or therapeutic targets.

## Introduction

Uterine leiomyoma is the most common benign tumor in women of reproductive age and represent an important public health issue. Patients usually present with abnormal uterine bleeding and sometimes complain of abdominal discomfort due to compression by the mass. The manifestations change throughout lifetime such as continuously growing or decreasing mass, irregular bleeding and rarely malignant transformation. Several treatment options exist ranging from observation or medical therapy including iron supplementation, oral contraceptive, and progesterone, uterine artery embolization and focused ultrasound, hysteroscopy or laparoscopic myomectomy to hysterectomy. Therefore, precise diagnosis, regular follow-up, and adequate treatment are important especially when patients show symptomatic changes related to uterine leiomyoma [1]. Clinicians should evaluate not only the symptoms but also associated conditions such as anemia, fertility preservation, and the possibility of malignancy before performing hysterectomy.

Although uterine leiomyoma is the most common cause of hysterectomy, its pathogenesis remains unclear, and hence the limited number of treatment options available for symptomatic patients. Recent studies compared the microRNA (miR) expression of uterine leiomyoma and myometrium and reported that a few specific miRs such as miR -150-5P [2], miR-29 family [3], and miR-let-7 [4] played a pathogenic role in the development of leiomyoma. miR expression profiles significantly differ among organs and tissues, and one miR has numerous targets. Although miR levels in cancer tissues are generally lower than those in normal tissues [5]. However, their clinical impact, specifically on disease severity, is not fully understood.

In our study, we analyzed miR in a sample set of 19 patients with leiomyoma using microarrays and real-time quantitative PCR (RT-qPCR). We observed distinct miR expression profiles between leiomyoma and adjacent myometrium and selected miRs showing prominent differences. We then selected two target molecules of selected miRs, Transforming Growth Factor Beta Receptor 2 (TGFBR2) and Insulin-like Growth Factor 2 mRNA Binding Protein 1 (IGF2BP1), for immunohistochemical analysis of corresponding tissue samples. The reported findings validate the role of miRs in mediating changes associated with the development and severity of leiomyoma.

## Materials and methods

### Study subjects and tissue specimens

The study enrolled 19 women who received surgical management for uterine leiomyoma (heavy menstrual bleeding, palpable mass) from May, 2018 to February, 2019 in a university hospital. The study received institutional review board approval from Bucheon St. Mary’s Hospital, The Catholic University of Korea (HCI8SESI0037), and all participants provided written informed consent. We conducted this study under the amended Declaration of Helsinki on the ethical conduct of research involving human subjects. Clinical data were analyzed through chart review, and the association between miR and cytokine was investigated.

Uterine tissues were collected from leiomyoma mass and adjacent (within 2 cm) myometrial tissues within 1 hour of surgery after examine by gynecologist and pathologist. miR was extracted from RNA in later-preserved tissues using a miRNeasy Mini Kit (Qiagen, Hilden, Germany) at -80°C, following the manufacturer’s protocol. RNA purity and integrity were evaluated by ND-1000 Spectrophotometer (NanoDrop, Wilmington, DE, USA) and Agilent 2100 Bioanalyzer (Agilent Technologies, Palo Alto, CA, USA).

### miR profiling using array

The miRs of 10 leiomyoma masses and 6 myometrial tissues were profiled using the Affymetrix Genechip miR 4.0 array according to the manufacturer’s protocol. RNA samples (130 ng) were labeled with the FlashTag™ Biotin RNA Labeling Kit (Genisphere, Hatfield, PA, USA). The labeled RNA was quantified, fractionated and hybridized to the miR microarray according to the standard procedures provided by Macrogen Inc. (Seoul, Korea). The labeled RNA was heated to 99°C for 5 min and then to 45°C for 5 min. RNA-array hybridization was performed via agitation at 60 rotations per min for 16 h at 48°C on an Affymetrix Hybridization oven. The chips were washed and stained using a Genechip Fluidics Station 450 (Affymetrix, Santa Clara, CA, USA). The chips were then scanned with an Affymetrix GCS 3000 scanner (Affymetrix). Signal values were computed using the Affymetrix^®^ GeneChip™ Command Console software. Raw data were extracted automatically via Affymetrix data extraction protocol using the software provided by Affymetrix GeneChip^®^ Command Console^®^ Software (AGCC). The CEL files were imported and miR level RMA+DABG were analyzed. All analyses and results were exported using Affymetrix^®^ Power Tools (APT) Software. After normalization, significant differentially expressed miRs were identified through Volcano Plot filtering between the two experimental groups. Finally, hierarchical clustering was performed for distinct miR expression profiling with at least 3.0-fold expression and *P* <0.05 of the samples.

### miR quantification using reverse transcription quantitative PCR (RT-qPCR)

RT-qPCR was performed to validate the expression of miRs using 38 samples including 19 pairs of myometrium and leiomyoma (S1 Fig). After a database search and literature review, miR-181a-5p [6–8], miR-127-3p [9–11], miR-28-3p [12], miR-30b-5p [13,14] and let-7c-5p [15,16] were selected for further validation (S1 Table). MicroRNA TaqMan^®^ Reverse Transcription Kit and TaqMan MicroRNA Assays were used (Applied Biosystems, Foster City,CA, USA). U6 small nuclear 2 (RNU6b) was selected to normalize miR expression levels. RT-qPCR was performed using the Lightcycler 480 PCR system (Roche, Basel, Switzerland). PCR reaction mixtures containing 10 ng DNA, 20X Taqman microRNA assay, 2X Taqman master mix (Applied Biosystems) were prepared and reactions were performed under the following conditions: 1 cycle of polymerase activation at 95°C for 10 min; 40 cycles of denaturation at 95°C for 15 sec, followed by annealing/extension at 60°C for 1 min. Results were analyzed using Lightcycler 480 instrument software 1.2 (Roche). Each experiment was performed in duplicate.

### Immunohistochemistry (IHC) of TGFBR2 and IGF2BP1

IHC was performed to evaluate the expression of TGFBR2 and IGF2BP1 in 16 leiomyoma tissues. Tissues were fixed in formalin and embedded in paraffin, and 3-μm-thick paraffin sections were obtained. IHC was performed using an automated immunohistochemical stainer (Ventana Medical Systems, Inc., Tucson, AZ, USA) according to the manufacturer’s protocol. The sections were deparaffinized and pretreated with a cell-conditioning solution (CC1, Ventana), followed by UV irradiation to abrogate the endogenous hydroperoxidase activity. The primary antibodies were diluted in Dako antibody diluent (Dako Cytomation, Glostrup, Denmark) with background-reducing components and used under the following dilutions: TGFBR2 (1:100, ab61213, Abcam, Franklin Lakes, NJ, USA) and IGF2BP1 (1:100, ab82968, Abcam,). The sections were incubated with primary antibodies at room temperature for 32 min, and hybridized with HRP-conjugated secondary antibody (Ventana) for 8 min. The reaction was developed with diaminobenzidine (DAB; Dako) for 5 min and the slides were counterstained with hematoxylin II (Ventana) for 4 min and bluing reagent (Ventana) for 4 min. The sections were observed under light microscope (BX50, Olympus, Japan).

### Statistical analysis

The comparative analysis of test and control samples was carried out using an independent t-test and fold change was determined, based on the null hypothesis stating that no difference exists among groups. False discovery rate (FDR) was controlled by adjusting the P value using Benjamini-Hochberg algorithm. All statistical tests and visualization of differentially expressed genes were conducted using R statistical language 3.3.3.

In real-time PCR, statistical analysis of the association between miR expression and different tumor histotypes was conducted using the non-parametric Mann-Whitney test. The Tukey’s multiple comparisons test was used to evaluate the differences in miR expression according to clinicopathological parameters. Pearson’s chi-square test was utilized to analyze the relationship between parametric data. Pearson’s correlation was performed for the quantitative analysis of size of leiomyoma and miR expression. All statistical analyses were performed using SPSS Statistics for Windows, Version 20.0 (IBM Corp., Amonk, NY, USA). *P* <0.05 was considered to indicate a statistically significant difference.

## Results

### Patient’s characteristics

The clinical characteristics of 19 participants are summarized in Table 1. The median age was 46 years and BMI was 22.9 ± 2.7 kg/m^2^. Irregular menstruation and heavy menstrual bleeding were reported by six (31.6%) and eight (42.1%) participants, respectively. Indications for surgery were mass effects including pressure symptoms and/or palpable mass (n = 8), heavy menstrual bleeding (n = 8) and both symptoms (n = 3). The mean size of the uterine leiomyoma was 7.3 ± 3.7 cm (2–15 cm) in its largest diameter. Eight patients (42.1%) received oral iron supplementation and three patients (15.8%) had a history of transfusion of packed red cells (PRC) due to anemia. Total laparoscopic hysterectomy was performed in 18 patients and myomectomy was conducted in 1 patient.

**Table 1:** Patient characteristics.

Variables		n = 19
Age (years)		46 (40–50)
Height (cm)		158.0 ± 4.8
Weight (kg)		57.5 ± 7.6
BMI (kg/m^2^)		22.9 ± 2.7
Parity		2 (0–3)
Irregular menstruation		31.6% (n = 6)
Dysmenorrhea		
	No	3 (15.8%)
	Mild	10 (52.6%)
	Moderate	5 (26.3%)
	Severe	1 (5.3%)
Symptom of uterine leiomyoma		
	Heavy menstrual bleeding	8 (42.1%)
	Mass effect (palpable mass)	8 (42.1%)
	Both symptoms	3 (15.8%)
Size of leiomyoma (cm)		7.3 ± 3.7 (2–15)
Hematologic test		
	Hgb (g/dL)	11.3 ± 2.5 (4.9–14.3)
	Hct (%)	34.4 ± 6.8 (18.8–43.2)
Oral iron supplement		42.1% (n = 8)
History of transfusion of PRC		15.8% (n = 3)

Data are expressed as median (range) or mean ± standard deviation.

BMI, body mass index; Hgb, hemoglobin; Hct, hematocrit; PRC, packed red cells.

### miR microarray analysis

**Fig 1:**
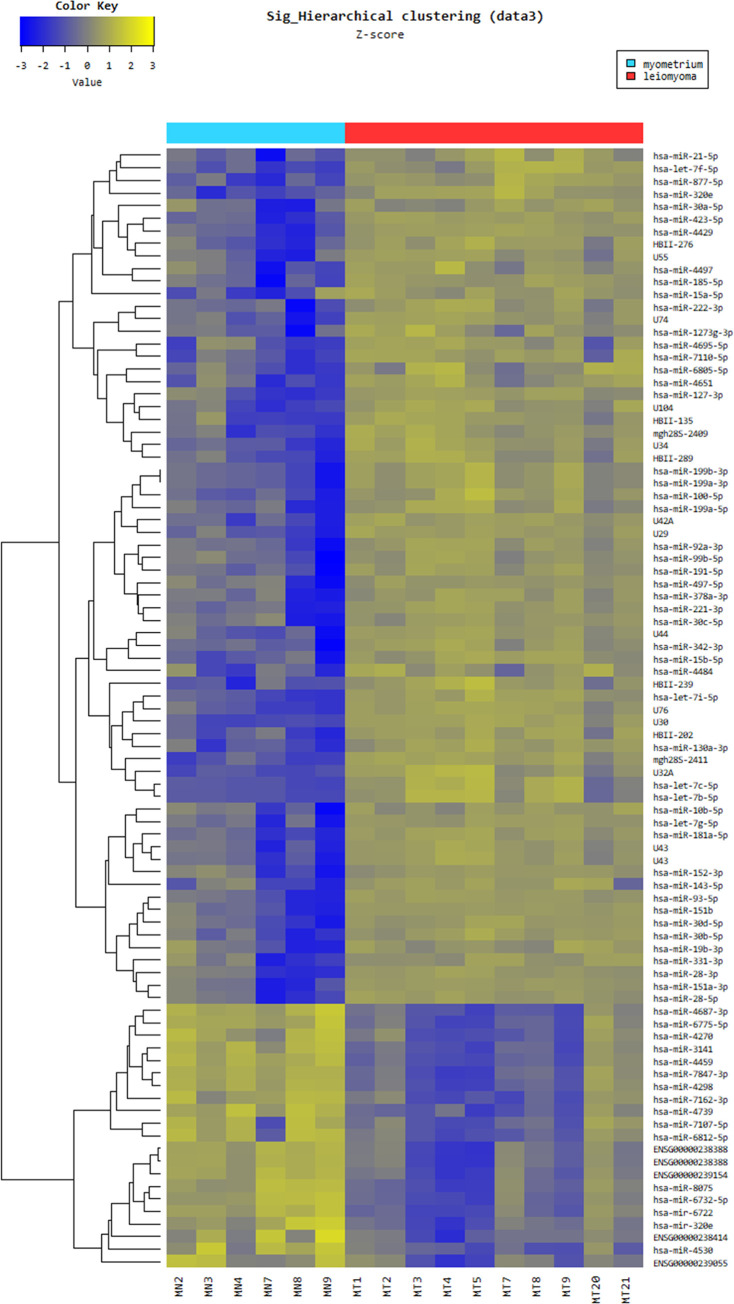
Differential expression of miRs between leiomyoma (n = 10) and myometrium (n = 6). Yellow color indicates high relative expression and blue color denotes low relative expression. miR with expression fold change >3.0 and *P* <0.05 was considered statistically significant.

**Fig 2:**
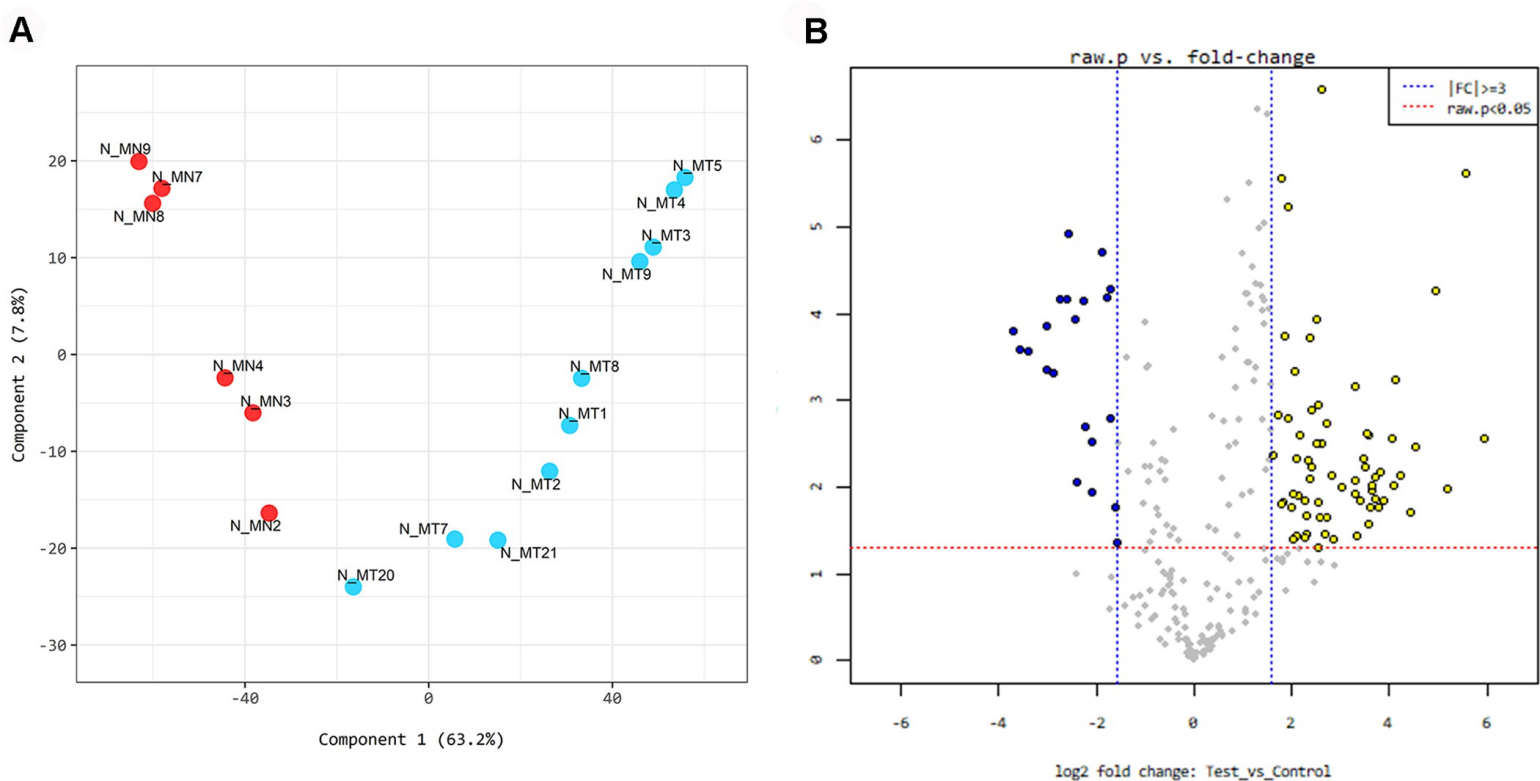
Multidimensional scaling (MDS) plot (A) and volcano plot (B) of the miR microarray analysis. (A) MDS plot shows similarity of miRNA profiles in leiomyoma (MT) and adjacent myometrium (MN). The dots represent samples colored by group. (B) Volcano plots of aberrantly expressed miRNAs in leiomyoma. The vertical lines correspond to 1.5-fold up-regulation and down-regulation, respectively, and the horizontal line represents a P value of 0.05. Abbreviation: FC, fold change.

Hierarchical clustering analysis [Euclidean method, complete linkage] was performed using the miR profiles of 10 leiomyomas and 6 samples of myometrium. MiR profile distinguished leiomyoma from myometrium and both were divided into two subcategories (Fig 1). A volcano plot was generated to visualize the differential expression of the two conditions and a multidimensional scaling plot was generated to represent the relationship between objects differing in miR data, as shown in Fig 2. To identify the most significant candidates, miRs with at least 3.0-fold expression variation were selected (S2 Table). A total of 89 miRs were expressed differentially between leiomyoma and myometrium (*P* <0.05) and among them 43 miRs showed FDR <0.05. Compared with the myometrium, the leiomyoma showed that 26 of the 43 miRs were up-regulated and 17 miRs were down-regulated, according to the criteria. The miR DB expressed differentially between leiomyoma and myometrium included 14 up-regulated miRs including hsa-miR-181a-5p, hsa-miR-4429, hsa-miR-423-5p, hsa-miR-7110-5p, hsa-miR-320e, hsa-miR-877-5p, hsa-let-7i-5p, hsa-let-7b-5p, hsa-miR-130a-3p, hsa-let-7f-5p, hsa-let-7c-5p, hsa-miR-100-5p, hsa-miR-199a-3p and hsa-miR-199b-3p, and 14 down-regulated miRs including hsa-miR-4270, hsa-miR-4739, hsa-miR-6775-5p, hsa-miR-4687-3p, hsa-miR-4530, hsa-miR-320e, hsa-miR-3141, hsa-miR-7847-3p, hsa-miR-4459, hsa-miR-4298, hsa-miR-6722, hsa-miR-7162-3p, hsa-miR-8075 and hsa-miR-6732-5p.

### RT-qPCR of miR expression

**Fig 3:**
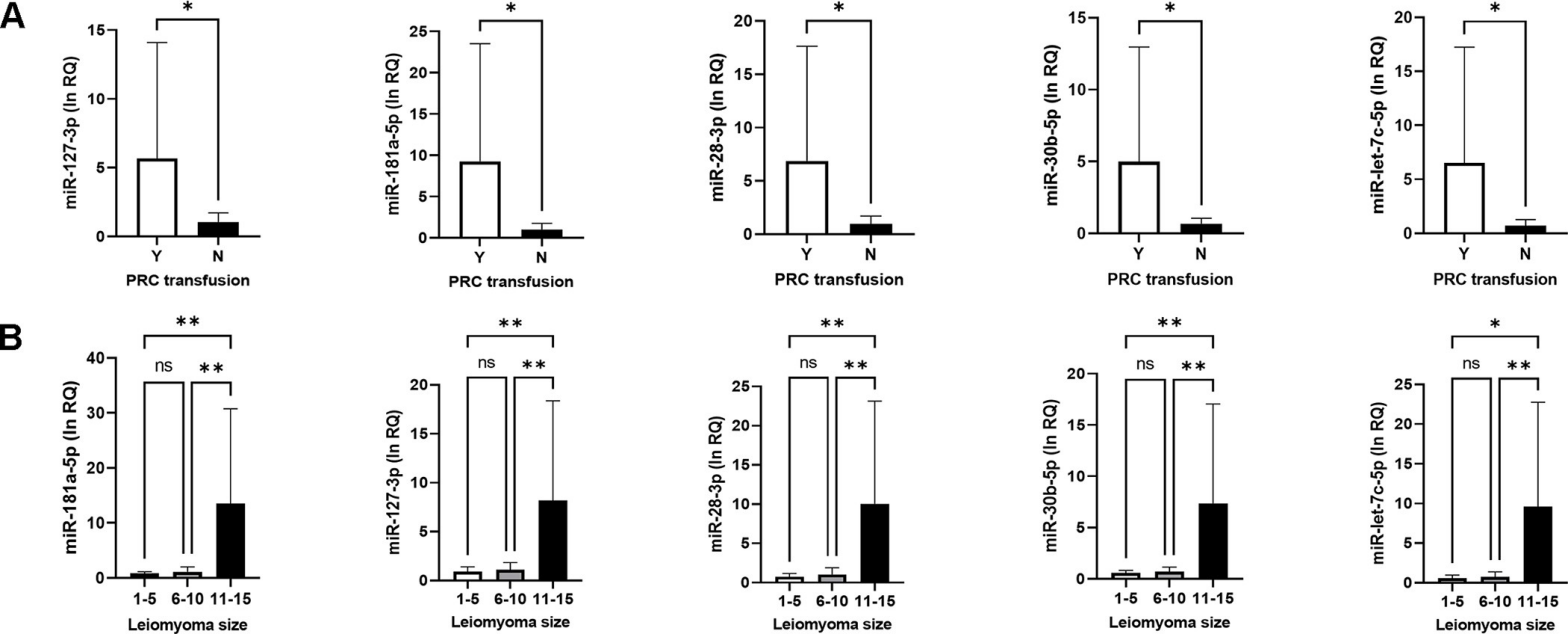
Comparative analysis of miR expression using RT-qPCR. All five miRs show significantly higher expression in (A) patients (n = 3) who had history of PRC transfusion due to anemia (Y) compared with those without (N) and (B) large leiomyoma (≥ 11 cm, n = 2). Results are presented as mean ± SD calculated using data obtained from independent samples of 19 leiomyomas and adjacent myometrial tissue.

Five selected miRs (miR-181a-5p, miR-127-3p, miR-28-3p, miR-30b-5p, and miR-let-7c) were expressed higher in leiomyoma than in myometrium. However, no statistically significant difference existed between the two groups due to high variation among samples (S2 Fig). We then compared the expression according to clinical characteristics including size of leiomyoma, delivery type, history of transfusion due to anemia, and iron supplementation therapy. Their relative expression levels were correlated with the size of leiomyoma (n = 16, *P* < 0.05, S3 Table). Leiomyoma samples from patients (n = 3) with a history of PRC transfusion due to anemia showed higher expression levels of the five miRs (*P* < 0.05; Fig 3A).We also found that miR-181a-5p, 127-3p, 28-3p, 30b-5p and let-7c-5p levels were significantly higher in large leiomyoma (≥ 11 cm, n = 2) than in leiomyoma measuring less than 11 cm (n = 14, *P* < 0.05, Fig 3B).

### Clinical significance of TGFBR2 and IGF2BP1 expression

**Fig 4:**
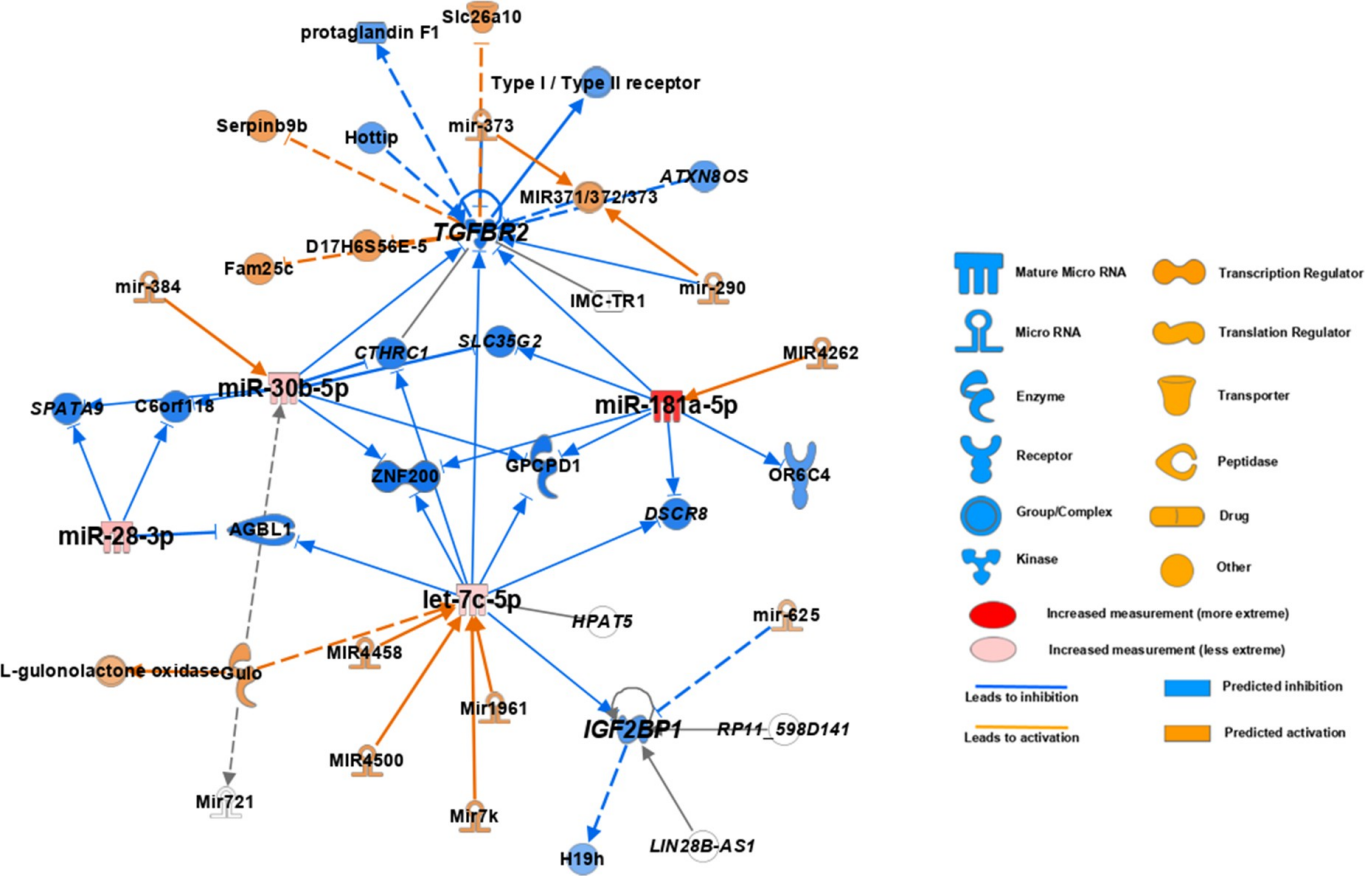
The association of differentially expressed miRs with TGFBR2 and IGF2BP1 analyzed using ingenuity pathway analysis.

We screened the predictable target molecules of differentially expressed miRs via the TargetScan (https://www.targetscan.org/) and miRDB (http://mirdb.org) and selected TGFBR2 and IGF2BP1, which were recurrently nominated. We also reviewed previous studies [3,17,18] revealing the association of miRs and selected two target molecules. In addition, we confirmed their association using the Ingenuity^®^ Pathway Analysis (IPA; QIAGEN Inc.) (Fig 4). IHC was successfully performed for two targets in 16 samples. We found that TGFBR2 and IGF2BP1 were positively stained in 13 (81%) and 10 (62.5%) leiomyoma tissues; however, neither of them was stained in the adjacent myometrium (S3 Fig). IGF2BP1 was more frequently positive in patients with ≥ 6 cm leiomyoma, irregular menstruation and mass effect. TGFBR2 was more frequently positive in patients with ≥ 6 cm leiomyoma and mass effect (Table 2). The mean expression levels of miR-181a-5p, 127-3p, 28-3p, 30b-5p and let-7c-5p were higher in patients with IGF2BP1 and TGFBR2-positive leiomyoma. However, no statistical significant difference was found between the two groups (S4 Fig).

**Table 2:** Comparison of IGF2BP1 and TGFBR2 expression.

	IGF2BP1			TGFBR2		
	Positive	Negative	*P*	Positive	Negative	*P*
Number of patients	10	6		13	3	
Leiomyoma size (≥ 6 cm)	80%	16.7%	0.013	69.2%	0%	0.029
Irregular menstruation	50.0%	0%	0.037			
Mass effect	80.0%	16.7%	0.013	69.2%	0%	0.029

Data describe median (range) or mean ± standard deviation.

TGFBR2, Transforming Growth Factor Beta Receptor 2; IGF2BP1, Insulin-like Growth Factor 2 mRNA Binding Protein 1.

## Discussion

We investigated the pathogenesis of leiomyoma based on miR expression profile and identified several significant miRs and their interaction with clinical findings. Differentially expressed miRs in leiomyoma were selected compared with those in adjacent myometrium in order to provide additional evidence of pathogenesis. Previous studies also revealed that a subset of miRs were significantly dysregulated in leiomyomas compared with matched myometrial samples [19–21] and many of these miRs were also associated with other neoplasms, indicating their general role in tumorigenesis [18]. These miRs are crucial regulators of physiological events including cellular proliferation, apoptosis, extracellular matrix turnover, angiogenesis and inflammation of uterine leiomyoma [22,23]. Recent studies reported that these miRs act as tumor suppressors and anti-apoptotic mediators (let-7 family, miR-17-92 cluster, miR-372-373, miR-155-BIC, miR 15/16, miR-20, miR-21, miR-26a), and play a role in differentiation and hypertrophy (let-7 family, miR-181b, miR-21), inflammation (miR-125, miR-155) and in tissue remodeling/extra-ECM turnover (miR-21, miR-192, miR-206, miR-1, miR-133a) [2,4,22,23].

We measured the expression of 5 up-regulated miRs in leiomyoma including miR-181a-5p, 127-3p, 28-3p, 30b-5p, and let-7c-5p using RT-qPCR, and found their interaction with the size of leiomyoma. The expression of miR-181a-5p and 127-3p strongly up-regulated during myoblast differentiation [6,9,11]. miR-181a is recognized as a senescence-associated miR and a potent negative regulator of Akt pathway [24]. It also affected the expression of insulin-like growth factor binding proteins (IGFBP), which might be of some importance in leiomyomas [7]. miR-127-3p is expressed at significantly higher level in proliferating rather than differentiating cells [10]. The miR-28-3p is a crucial regulator of myogenic differentiation of rhabdomyosarcoma cells and myoblasts [12]. The expression of miR-30b-5p was associated with differentiation of both skeletal and vascular smooth muscles via interaction with MBNL1 and/or MBNL2 [13,14]. Let-7 is one of the most widely studied miRs and let-7c-5p is important for cell growth, proliferation and inflammation [25]. Genetic alterations, cellular transformation of myometrial cells, ovarian steroids may affect the expression of Let-7 family and their aberrant expression alter the stability of their target gens (vascular endothelial growth factor(VEGF), fibroblast growth factor(FGF), interleukin(IL),TGF, tumor necrosis factor(TNF) et al).The product of some of these genes may in turn regulate the expression of miR, which through a feedback mechanism influence various cellular activities including tissue turnover, cell-cycle progression, hypertrophy, angiogenesis, and inflammation resulting in tissue fibrosis, a characteristic of leiomyoma during growth[26]. Let-7 expression was significantly decreased in leiomyosarcoma tissues, which may contribute to the malignant transformation of leiomyoma accompanied by overexpression of HMGA2 [15,16].Therefore, we postulated that the up-regulated miRs modify target molecules in addition to proliferation and differentiation of muscle cells and were associated with the development and growth of leiomyoma.

Although the expression and possible regulatory functions on miRs in leiomyoma with particular emphasis on the expression of their selective target genes whose products effect various cellular activities critical to pathogenesis of leiomyomas, there are few studies on the definite evidence for the association. We investigated the expression of two target molecules of those miRs via IHC. Leiomyoma tissues but not the adjacent myometrium stained positive for TGFBR2 and IGF2BP1. TGFBR2 is a receptor of a secreted polypeptide TGF-beta2, which regulates leiomyoma growth [27].The IGF2BP1, an oncofetal RNA-binding protein, has been identified to play an important role in cell proliferation and growth of normal tissues and tumor tissues, as well as tumor cell adhesion, apoptosis, migration, and invasion [28]. Studies have demonstrated that fibroblasts and smooth muscle cells from leiomyoma tissue not only increase the expression and secretion of growth factors including TGF-beta and IGF, but also up-regulate their corresponding receptors [29–31]. Notably, the overexpression of both proteins was associated with leiomyoma size and mass effect. Therefore, we strongly suspected that such changes in miR up-regulated TGFBR2 and IGF2BP1 in leiomyoma tissue and affected its growth.

Although we have investigated the pathogenic role of miRs in leiomyoma, the study has some limitations. First, the number of cases was relatively small to demonstrate the clinical significance other than leiomyoma size. Second, no direct interaction between miRs and protein expression was identified. Previous studies suggested that increased miRs such as miR-181a-5p, 28-3p and let-7 family may represent new therapeutic targets in various diseases [8,12,32]. Further studies are needed to validate the significance of the miRs as diagnostic or therapeutic targets.

Taken together, the results demonstrated that miRs played a significant role in the pathogenesis of leiomyoma. Particularly, the five miRs including miR-181a-5p, 127-3p, 28-3p, 30b-5p and let-7c-5p, and their target molecules including TGFBR2 and IGF2BP1, were up-regulated in leiomyoma tissues. Additionally, their expression was associated with leiomyoma size and size effect. These findings are significant in understanding of miRs and their role as a potential indicator of leiomyoma development and disease progression.

## Supporting information

S1 Fig Comparison of miR expression analyzed by RT-qPCR. All five miRs show higher expression level in leiomyoma compared to in adjacent myometrium. However, there are no statistic significance due to high variation among limited number of samples.
(DOCX)

S2 Fig Representative images of immunohistochemical staining. IGF2BP1 (upper) and TGFBR2 (lower) with positive (left) and negative (right) staining pattern. Images were obtained with a Carl Zeiss Microscopy GmbH (Carl Zeiss, Jena, Germany) equipped with a ProgRes MF camera (JENOPTIK, Jena, Germany). Original magnification, 400×.
(DOCX)

S3 Fig Comparison of miR expression according to the staining pattern of TGFBR and IGF2BP1. All five miRs show higher expression level in leiomyoma compared to in adjacent myometrium. However, there are no statistical significance due to high variation among limited number of samples.
(DOCX)

S4 Fig Comparison of miR expression according to the staining pattern of TGFBR2 and IGF2BP1. All five miRs show higher expression level in leiomyoma compared to in adjacent myometrium. However, there are no statistical significance due to high variation among limited number of samples.
(DOCX)

S1 Table Taqman miR assays used in this study. (DOCX)

S2 Table miRs with at least 3 fold expression changes. (DOCX)

S3 Table Correlation between miR expressions with size of leiomyoma. (DOCX)

S1 Data (XLSX)

## References

[1] Berek JS, Novak E. Berek and Novak’s Gynecology. 16th ed. Philadelphia: Lippincott Williams & Wilkins; 2019.

[2] Lee JH, Choi YS, Park JH, Kim H, Lee I, Won YB, et al. MiR-150-5p may contribute to pathogenesis of human leiomyoma via regulation of the Akt/p27(Kip1) pathway in vitro. Int J Mol Sci. 2019; 20:2684. 10.3390/ijms20112684 PMC6601023 31159158

[3] Islam MS, Ciavattini A, Petraglia F, Castellucci M, Ciarmela P. Extracellular matrix in uterine leiomyoma pathogenesis: a potential target for future therapeutics. Hum Reprod Update. 2018; 24:59–85. doi: 10.1093/humupd/dmx032 29186429

[4] Karmon AE, Cardozo ER, Rueda BR, Styer AK. MicroRNAs in the development and pathobiology of uterine leiomyomata: does evidence support future strategies for clinical intervention? Hum Reprod Update. 2014; 20:670–687. doi: 10.1093/humupd/dmu017 24706045

[5] Yanokura M, Banno K, Kobayashi Y, Kisu I, Ueki A, Ono A, et al. MicroRNA and endometrial cancer: Roles of small RNAs in human tumors and clinical applications (Review). Oncol Lett. 2010; 1:935–940. doi: 10.3892/ol.2010.173 22870090 PMC3412506

[6] Wei Y, Tao X, Xu H, Chen Y, Zhu L, Tang G, et al. Role of miR-181a-5p and endoplasmic reticulum stress in the regulation of myogenic differentiation. Gene. 2016; 592:60–70. doi: 10.1016/j.gene.2016.07.056 27461948

[7] Wu L, Song WY, Xie Y, Hu LL, Hou XM, Wang R, et al. miR-181a-5p suppresses invasion and migration of HTR-8/SVneo cells by directly targeting IGF2BP2. Cell Death Dis. 2018; 9:16. doi: 10.1038/s41419-017-0045-0 29339719 PMC5833820

[8] Chen G, Shen ZL, Wang L, Lv CY, Huang XE, Zhou RP. Hsa-miR-181a-5p expression and effects on cell proliferation in gastric cancer. Asian Pac J Cancer Prev. 2013; 14:3871–3875. doi: 10.7314/apjcp.2013.14.6.3871 23886199

[9] Zhai L, Wu R, Han W, Zhang Y, Zhu D. miR-127 enhances myogenic cell differentiation by targeting S1PR3. Cell Death & Disease. 2017; 8:e2707–e2707. doi: 10.1038/cddis.2017.128 28358363 PMC5386531

[10] Li J, Wang G, Jiang J, Zhang L, Zhou P, Ren H. MicroRNA-127-3p regulates myoblast proliferation by targeting Sept7. Biotechnol Lett. 2020; 42:1633–1644. doi: 10.1007/s10529-020-02906-0 32382971

[11] Li J, Wang G, Jiang J, Zhou P, Liu L, Zhao J, et al. Dynamical Expression of MicroRNA-127-3p in Proliferating and Differentiating C2C12 Cells. Asian-Australas J Anim Sci. 2016; 29:1790–1795. doi: 10.5713/ajas.15.0968 26954209 PMC5088429

[12] Skrzypek K, Nieszporek A, Badyra B, Lasota M, Majka M. Enhancement of myogenic differentiation and inhibition of rhabdomyosarcoma progression by miR-28-3p and miR-193a-5p regulated by SNAIL. Mol Ther Nucleic Acids. 2021; 24:888–904. doi: 10.1016/j.omtn.2021.04.013 34094709 PMC8141673

[13] Zhang B-W, Cai H-F, Wei X-F, Sun J-J, Lan X-Y, Lei C-Z, et al. miR-30-5p Regulates Muscle Differentiation and Alternative Splicing of Muscle-Related Genes by Targeting MBNL. International journal of molecular sciences. 2016; 17:182. doi: 10.3390/ijms17020182 26840300 PMC4783916

[14] Woo CC, Liu W, Lin XY, Dorajoo R, Lee KW, Richards AM, et al. The interaction between 30b-5p miRNA and MBNL1 mRNA is involved in vascular smooth muscle cell differentiation in patients with coronary atherosclerosis. International journal of molecular sciences. 2019; 21:11. doi: 10.3390/ijms21010011 31861407 PMC6982107

[15] Shi G, Perle MA, Mittal K, Chen H, Zou X, Narita M, et al. Let-7 repression leads to HMGA2 overexpression in uterine leiomyosarcoma. J Cell Mol Med. 2009; 13:3898–3905. doi: 10.1111/j.1582-4934.2008.00541.x 19602040 PMC4516537

[16] de Almeida BC, Dos Anjos LG, Uno M, Cunha IWd, Soares FA, Baiocchi G, et al. Let-7 miRNA’s Expression Profile and Its Potential Prognostic Role in Uterine Leiomyosarcoma. Cells. 2019; 8:1452. doi: 10.3390/cells8111452 31744257 PMC6912804

[17] Zavadil J, Ye H, Liu Z, Wu J, Lee P, Hernando E, et al. Profiling and functional analyses of microRNAs and their target gene products in human uterine leiomyomas. PloS one. 2010; 5:e12362–e12362. doi: 10.1371/journal.pone.0012362 20808773 PMC2927438

[18] Wang T, Zhang X, Obijuru L, Laser J, Aris V, Lee P, et al. A micro-RNA signature associated with race, tumor size, and target gene activity in human uterine leiomyomas. Genes Chromosomes Cancer. 2007; 46:336–347. doi: 10.1002/gcc.20415 17243163

[19] Marsh EE, Steinberg ML, Parker JB, Wu J, Chakravarti D, Bulun SE. Decreased expression of microRNA-29 family in leiomyoma contributes to increased major fibrillar collagen production. Fertil Steril. 2016; 106:766–772. doi: 10.1016/j.fertnstert.2016.05.001 27233758 PMC5011009

[20] Pan Q, Luo X, Chegini N. Differential expression of microRNAs in myometrium and leiomyomas and regulation by ovarian steroids. J Cell Mol Med. 2008; 12:227–240. doi: 10.1111/j.1582-4934.2007.00207.x 18182067 PMC2730932

[21] Zavadil J, Ye H, Liu Z, Wu J, Lee P, Hernando E, et al. Profiling and functional analyses of microRNAs and their target gene products in human uterine leiomyomas. PLoS One. 2010; 5:e12362. doi: 10.1371/journal.pone.0012362 20808773 PMC2927438

[22] Chuang TD, Khorram O. Expression profiling of incRNAs, miRNAs, and mRNAs and their differential expression in leiomyoma using next-generation RNA sequencing. Reprod Sci. 2018; 25:246–255. doi: 10.1177/1933719117711265 28587571

[23] Gao Y, Dai M, Liu H, He W, Lin S, Yuan T, et al. Diagnostic value of circulating miR-21: An update meta-analysis in various cancers and validation in endometrial cancer. Oncotarget. 2016; 7:68894–68908. doi: 10.18632/oncotarget.12028 27655698 PMC5356598

[24] Xu X, Kim JJ, Li Y, Xie J, Shao C, Wei JJ. Oxidative stress-induced miRNAs modulate AKT signaling and promote cellular senescence in uterine leiomyoma. J Mol Med (Berl). 2018; 96:1095–1106. doi: 10.1007/s00109-018-1682-1 30097674 PMC6135677

[25] Horak M, Novak J, Bienertova-Vasku J. Muscle-specific microRNAs in skeletal muscle development. Dev Biol. 2016; 410:1–13. doi: 10.1016/j.ydbio.2015.12.013 26708096

[26] Luo X, Chegini N. The expression and potential regulatory functionof microRNAs in the pathogenesis of leiomyoma. Semin Reprod Med. 2008; 26:500–514. doi: 10.1055/s-0028-1096130 18951332 PMC2710997

[27] Chegini N, Luo X, Ding L, Ripley D. The expression of Smads and transforming growth factor beta receptors in leiomyoma and myometrium and the effect of gonadotropin releasing hormone analogue therapy. Mol Cell Endocrinol. 2003; 209:9–16. doi: 10.1016/j.mce.2003.08.007 14604812

[28] Bell JL, Wachter K, Muhleck B, Pazaitis N, Kohn M, Lederer M, et al. Insulin-like growth factor 2 mRNA-binding proteins (IGF2BPs): post-transcriptional drivers of cancer progression? Cell Mol Life Sci. 2013; 70:2657–2675. doi: 10.1007/s00018-012-1186-z 23069990 PMC3708292

[29] Leppert PC, Catherino WH, Segars JH. A new hypothesis about the origin of uterine fibroids based on gene expression profiling with microarrays. Am J Obstet Gynecol. 2006; 195:415–420. doi: 10.1016/j.ajog.2005.12.059 16635466 PMC4143906

[30] Tang XM, Dou Q, Zhao Y, McLean F, Davis J, Chegini N. The expression of transforming growth factor-beta s and TGF-beta receptor mRNA and protein and the effect of TGF-beta s on human myometrial smooth muscle cells in vitro. Mol Hum Reprod. 1997; 3:233–240. doi: 10.1093/molehr/3.3.233 9237249

[31] Moore AB, Yu L, Swartz CD, Zheng X, Wang L, Castro L, et al. Human uterine leiomyoma-derived fibroblasts stimulate uterine leiomyoma cell proliferation and collagen type I production, and activate RTKs and TGF beta receptor signaling in coculture. Cell Commun Signal. 2010; 8:10. doi: 10.1186/1478-811X-8-10 20537183 PMC2897788

[32] Gilles ME, Slack FJ. Let-7 microRNA as a potential therapeutic target with implications for immunotherapy. Expert Opin Ther Targets. 2018; 22:929–939. doi: 10.1080/14728222.2018.1535594 30328720 PMC6337723

