# Supplementary material for: MicroRNAs as potential indicators of the development and progression of uterine leiomyoma
Source: PLoS One. 2022 May 31;17(5):e0268793. doi: 10.1371/journal.pone.0268793 (PMC9154092; doi:10.1371/journal.pone.0268793)
Supplement: S1 Fig — All five miRs show higher expression level in leiomyoma compared to in adjacent myometrium. However, there are no statistic significance due to high variation among limited number of samples. (DOCX) [file pone.0268793.s001.docx]

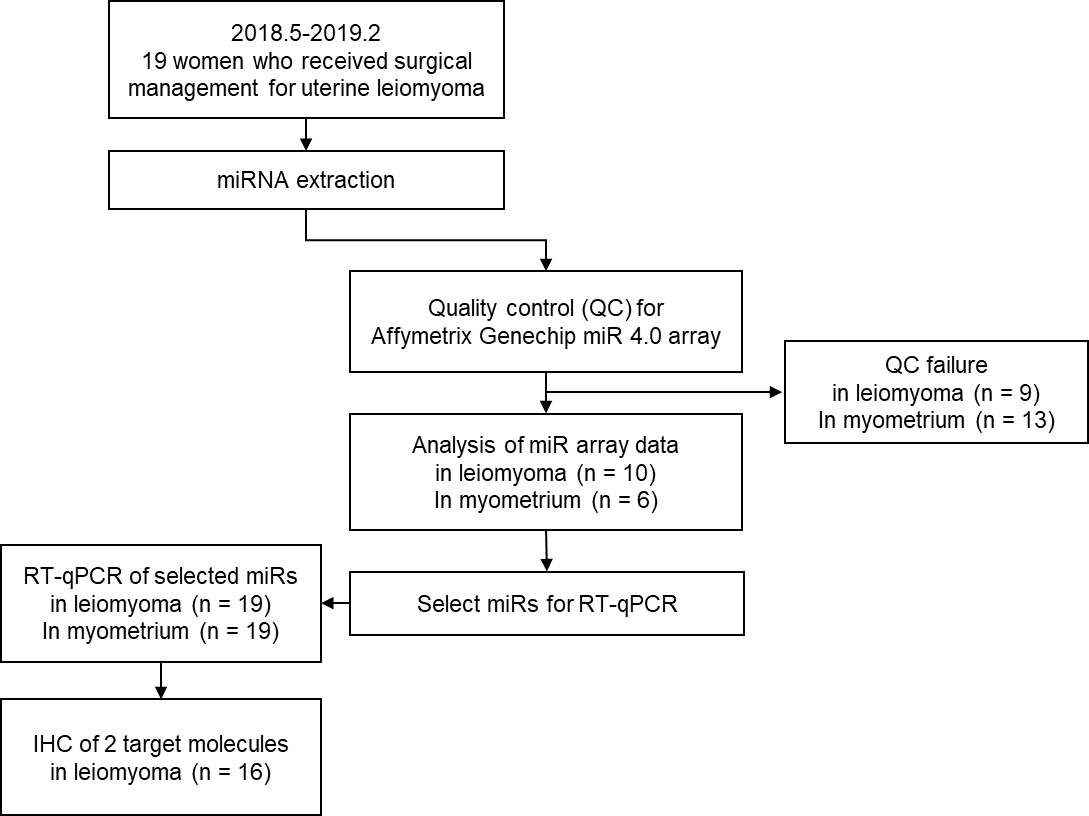


**S1 Fig. Flow chart showing the sample selection for experiments.** miR, miRNA; RT-qPCR, reverse transcription quantitative polymerase chain reaction; IHC, immunohistochemistry.
