# Supplementary material for: MicroRNAs as potential indicators of the development and progression of uterine leiomyoma
Source: PLoS One. 2022 May 31;17(5):e0268793. doi: 10.1371/journal.pone.0268793 (PMC9154092; doi:10.1371/journal.pone.0268793)
Supplement: S3 Fig — All five miRs show higher expression level in leiomyoma compared to in adjacent myometrium. However, there are no statistical significance due to high variation among limited number of samples. (DOCX) [file pone.0268793.s003.docx]

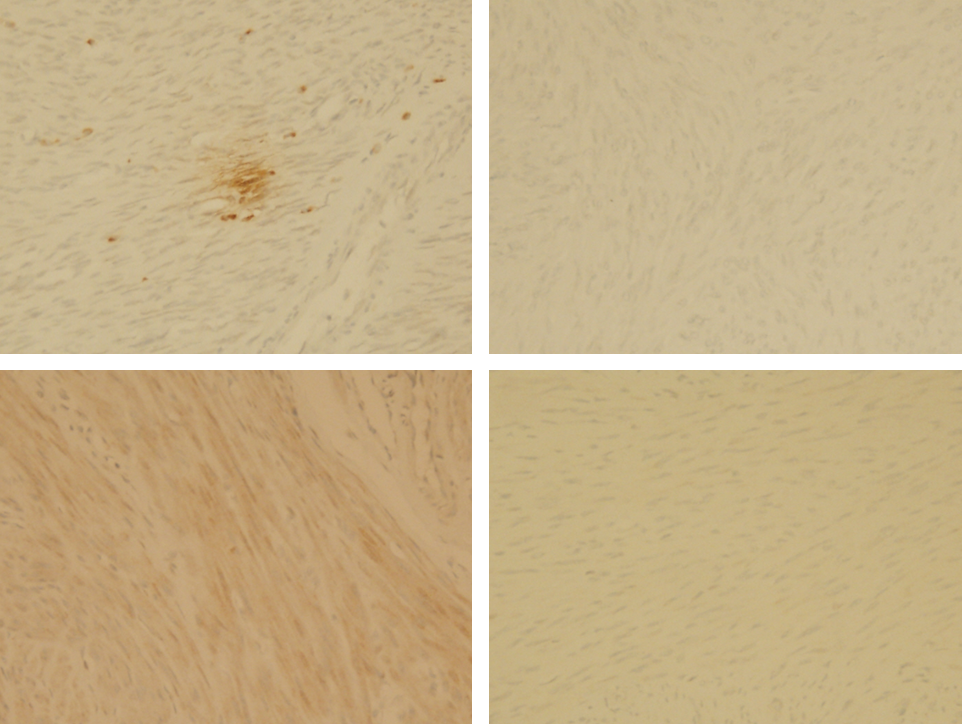


**S3 Fig. Representative images of immunohistochemical staining.** IGF2BP1, (upper) and TGFBR2 (lower) with positive (left) and negative (right) staining pattern. Images were obtained with a Carl Zeiss Microscopy GmbH (Carl Zeiss, Jena, Germany) equipped with a ProgRes MF camera (JENOPTIK, Jena, Germany). Original magnification, 400×.

IGF2BP1, Insulin-like Growth Factor 2 mRNA Binding Protein 1; TGFBR2, Transforming Growth Factor Beta Receptor 2
