# Supplementary material for: MicroRNAs as potential indicators of the development and progression of uterine leiomyoma
Source: PLoS One. 2022 May 31;17(5):e0268793. doi: 10.1371/journal.pone.0268793 (PMC9154092; doi:10.1371/journal.pone.0268793)
Supplement: S1 Table — (DOCX) [file pone.0268793.s005.docx]

**S1 Table. Taqman miR assays used in this study**

| **Assay name** | **Assay ID** | **Availability** | **Cat#** | **Species** |
| --- | --- | --- | --- | --- |
| hsa-miR-181a-5p | 000-480 | Inventoried | 4427975 | human |
| has-miR-127-3p | 000-452 | Inventoried | 4427975 | human |
| hsa-miR-28-3p | 002-446 | Inventoried | 4427975 | human |
| hsa-miR-30b-5p | 000-602 | Inventoried | 4427975 | human |
| has-let-7c-5p | 000-379 | Inventoried | 4427975 | human |
