# Supplementary material for: MicroRNAs as potential indicators of the development and progression of uterine leiomyoma
Source: PLoS One. 2022 May 31;17(5):e0268793. doi: 10.1371/journal.pone.0268793 (PMC9154092; doi:10.1371/journal.pone.0268793)
Supplement: S2 Table — (DOCX) [file pone.0268793.s006.docx]

**S2 Table. miRs with at least 3.0 fold expression changes.**

| **ProbeID** | **Test/Control.fc** | ***P*** | **FDR** | **Transcript ID (Array Design)** | **Alignments** | **Sequence Length** | **Sequence** | **miRDB TargetScan mirBase miRTarBase** |
| --- | --- | --- | --- | --- | --- | --- | --- | --- |
| 20500444 | 61.045428 | 0.0027937 | 0.0363378 | hsa-miR-181a-5p | chr1:198828237-198828259 (-) /// chr9:127454759-127454781 (+) | 23 | AACAUUCAACGCUGUCGGUGAGU | [hsa-miR-181a-5p](http://mirdb.org/cgi-bin/search.cgi?searchType=miRNA&searchBox=hsa-miR-181a-5p) |
| 20538159 | 47.265251 | 2.383E-06 | 0.0002412 | U30 | chr11:62621135-62621204 (-) | 70 | GTTTGTGATGACTTACATGGAATCTCGTTCGGCTGATGACTTGCTGTTGAGACTCTGAAATCTGATTTTC | --- |
| 20500771 | 35.976975 | 0.0103922 | 0.0888209 | hsa-miR-127-3p | chr14:101349372-101349393 (+) | 22 | UCGGAUCCGUCUGAGCUUGGCU | [hsa-miR-127-3p](http://mirdb.org/cgi-bin/search.cgi?searchType=miRNA&searchBox=hsa-miR-127-3p) |
| 20538300 | 30.942353 | 5.518E-05 | 0.0021852 | mgh28S-2411 | chr11:93464669-93464739 (-) | 71 | GATGTTATGATGATGGGCGAAATGTTCAACTGCTCTGAAGGGGCTGAATGAAAATGGCCTTTCTGAACATC | --- |
| 20518801 | 23.202875 | 0.0034813 | 0.0413352 | hsa-miR-4429 | chr2:11680778-11680797 (-) | 20 | AAAAGCUGGGCUGAGAGGCG | [hsa-miR-4429](http://mirdb.org/cgi-bin/search.cgi?searchType=miRNA&searchBox=hsa-miR-4429) |
| 20500159 | 21.739688 | 0.0191548 | 0.1286944 | hsa-miR-28-3p | chr3:188406622-188406643 (+) | 22 | CACUAGAUUGUGAGCUCCUGGA | [hsa-miR-28-3p](http://mirdb.org/cgi-bin/search.cgi?searchType=miRNA&searchBox=hsa-miR-28-3p) |
| 20500724 | 18.665165 | 0.007315 | 0.0707749 | hsa-miR-30b-5p | chr8:135812813-135812834 (-) | 22 | UGUAAACAUCCUACACUCAGCU | [hsa-miR-30b-5p](http://mirdb.org/cgi-bin/search.cgi?searchType=miRNA&searchBox=hsa-miR-30b-5p) |
| 20538249 | 17.462368 | 0.0005679 | 0.0118335 | U76 | chr1:173835773-173835852 (-) | 80 | GCCACAATGATGACAGTTTATTTGCTACTCTTGAGTGCTAGAATGATGAGGATCTTAACCACCATTATCTTAACTGAGGC | --- |
| 20504561 | 17.057079 | 0.0093885 | 0.0836946 | hsa-miR-151b | chr14:100575775-100575792 (-) | 18 | UCGAGGAGCUCACAGUCU | [hsa-miR-151b](http://mirdb.org/cgi-bin/search.cgi?searchType=miRNA&searchBox=hsa-miR-151b) |
| 20502123 | 16.481622 | 0.0027913 | 0.0363378 | hsa-miR-423-5p | chr17:28444113-28444135 (+) | 23 | UGAGGGGCAGAGAGCGAGACUUU | [hsa-miR-423-5p](http://mirdb.org/cgi-bin/search.cgi?searchType=miRNA&searchBox=hsa-miR-423-5p) |
| 20501287 | 14.620526 | 0.0140694 | 0.1087887 | hsa-miR-151a-3p | chr8:141742686-141742706 (-) | 21 | CUAGACUGAAGCUCCUUGAGG | [hsa-miR-151a-3p](http://mirdb.org/cgi-bin/search.cgi?searchType=miRNA&searchBox=hsa-miR-151a-3p) |
| 20534220 | 14.024933 | 0.0066465 | 0.0659845 | HBII-135 | chr17:16344540-16344612 (+) | 73 | AAATGATGAAATCACCCAAAATAGCTGGAATTACCGGCAGATTGTGTAGTGGTGAACCTATGGTTTTCTGAAG | --- |
| 20500713 | 13.857726 | 0.0167793 | 0.1191366 | hsa-let-7g-5p | chr3:52302352-52302373 (-) | 22 | UGAGGUAGUAGUUUGUACAGUU | [hsa-let-7g-5p](http://mirdb.org/cgi-bin/search.cgi?searchType=miRNA&searchBox=hsa-let-7g-5p) |
| 20500173 | 13.028517 | 0.0077215 | 0.0737011 | hsa-miR-93-5p | chr7:99691438-99691460 (-) | 23 | CAAAGUGCUGUUCGUGCAGGUAG | [hsa-miR-93-5p](http://mirdb.org/cgi-bin/search.cgi?searchType=miRNA&searchBox=hsa-miR-93-5p) |
| 20500787 | 13.018590 | 0.0134688 | 0.1051127 | hsa-miR-185-5p | chr22:20020676-20020697 (+) | 22 | UGGAGAGAAAGGCAGUUCCUGA | [hsa-miR-185-5p](http://mirdb.org/cgi-bin/search.cgi?searchType=miRNA&searchBox=hsa-miR-185-5p) |
| 20500424 | 12.459478 | 0.0106413 | 0.0901047 | hsa-miR-30d-5p | chr8:135817162-135817183 (-) | 22 | UGUAAACAUCCCCGACUGGAAG | [hsa-miR-30d-5p](http://mirdb.org/cgi-bin/search.cgi?searchType=miRNA&searchBox=hsa-miR-30d-5p) |
| 20538180 | 12.440044 | 0.0095484 | 0.0842358 | U43 | chr22:39715057-39715118 (-) | 62 | CACAGATGATGAACTTATTGACGGGCGGACAGAAACTGTGTGCTGATTGTCACGTTCTGATT | --- |
| 20500126 | 12.268693 | 0.0169524 | 0.1200188 | hsa-miR-15a-5p | chr13:50623303-50623324 (-) | 22 | UAGCAGCACAUAAUGGUUUGUG | [hsa-miR-15a-5p](http://mirdb.org/cgi-bin/search.cgi?searchType=miRNA&searchBox=hsa-miR-15a-5p) |
| 20538123 | 11.873645 | 0.0024813 | 0.0336617 | U104 | chr17:62223438-62223517 (+) | 80 | GGCCTGCTGTGATGACATTCCAATTAAAGCACGTGTTAGACTGCTGACGCGGGTGATGCGAACTGGAGTCTGAGCCTGCC | --- |
| 20501243 | 11.845334 | 0.0264173 | 0.1595534 | hsa-miR-378a-3p | chr5:149112430-149112450 (+) | 22 | ACUGGACUUGGAGUCAGAAGGC | [hsa-miR-378a-3p](http://mirdb.org/cgi-bin/search.cgi?searchType=miRNA&searchBox=hsa-miR-378a-3p) |
| 20538299 | 11.681392 | 0.0023585 | 0.0324789 | mgh28S-2409 | chr11:93466394-93466466 (-) | 73 | GTTCAGATGATGAATTTAACTGTTCAACTGCTGAATGATAACGGGCATGAACTAAAACTTAATTCTGACAGAG | --- |
| 20538181 | 11.463471 | 0.0057467 | 0.0600546 | U43 | chr22:39715057-39715118 (-) | 62 | CACAGATGATGAACTTATTGACGGGCGGACAGAAACTGTGTGCTGATTGTCACGTTCTGATT | --- |
| 20500718 | 11.079263 | 0.0046872 | 0.0518842 | hsa-miR-15b-5p | chr3:160122395-160122416 (+) | 22 | UAGCAGCACAUCAUGGUUUACA | [hsa-miR-15b-5p](http://mirdb.org/cgi-bin/search.cgi?searchType=miRNA&searchBox=hsa-miR-15b-5p) |
| 20500158 | 10.532833 | 0.0143109 | 0.1092766 | hsa-miR-28-5p | chr3:188406582-188406603 (+) | 22 | AAGGAGCUCACAGUCUAUUGAG | [hsa-miR-28-5p](http://mirdb.org/cgi-bin/search.cgi?searchType=miRNA&searchBox=hsa-miR-28-5p) |
| 20500758 | 10.070406 | 0.0358389 | 0.194641 | hsa-miR-152-3p | chr17:46114540-46114560 (-) | 21 | UCAGUGCAUGACAGAACUUGG | [hsa-miR-152-3p](http://mirdb.org/cgi-bin/search.cgi?searchType=miRNA&searchBox=hsa-miR-152-3p) |
| 20538182 | 9.838438 | 0.0083513 | 0.0772829 | U44 | chr1:173835104-173835166 (-) | 63 | CCTGGATGATGATAAGCAAATGCTGACTGAACATGAAGGTCTTAATTAGCTCTAACTGACTAA | --- |
| 20538165 | 9.817200 | 0.0006812 | 0.0134046 | U34 | chr19:49994164-49994229 (+) | 66 | CGTCCATGATGTTCCGCAACTACCTACATTGTTTGATCCTCATGAAAGCAGCACTGGCTGAGACGC | --- |
| 20500484 | 9.744391 | 0.0120827 | 0.0980145 | hsa-miR-221-3p | chrX:45605608-45605630 (-) | 23 | AGCUACAUUGUCUGCUGGGUUUC | [hsa-miR-221-3p](http://mirdb.org/cgi-bin/search.cgi?searchType=miRNA&searchBox=hsa-miR-221-3p) |
| 20500751 | 8.086010 | 0.0100783 | 0.0872695 | hsa-miR-143-5p | chr5:148808507-148808528 (+) | 22 | GGUGCAGUGCUGCAUCUCUGGU | [hsa-miR-143-5p](http://mirdb.org/cgi-bin/search.cgi?searchType=miRNA&searchBox=hsa-miR-143-5p) |
| 20500422 | 7.239122 | 0.0392265 | 0.2053506 | hsa-miR-30c-5p | chr1:41222972-41222994 (+) /// chr6:72086706-72086728 (-) | 23 | UGUAAACAUCCUACACUCUCAGC | [hsa-miR-30c-5p](http://mirdb.org/cgi-bin/search.cgi?searchType=miRNA&searchBox=hsa-miR-30c-5p) |
| 20538246 | 7.071064 | 0.0073563 | 0.0708292 | U74 | chr1:173836812-173836883 (-) | 72 | CTGCCTCTGATGAAGCCTGTGTTGGTAGGGACATCTGAGAGTAATGATGAATGCCAACCGCTCTGATGGTGG | --- |
| 20538204 | 6.537195 | 0.0222137 | 0.1432096 | U55 | chr1:45241537-45241610 (+) | 74 | GTGTATGATGACAACTCGGTAATGCTGCATACTCCCGAGTGCGCGGTGGGGAAGCCAACCTTGGAGAGCTGAGC | --- |
| 20534229 | 6.510467 | 0.0018373 | 0.0269921 | HBII-202 | chr16:89627838-89627909 (+) | 72 | CGCGTGATGACATTCTCCGGAATCGCTGTACGGCCTTGATGAAAGCACATTTGAACCCTTTTCCATCTGATT | --- |
| 20501293 | 6.475957 | 0.0342327 | 0.1896331 | hsa-miR-331-3p | chr12:95702256-95702276 (+) | 21 | GCCCCUGGGCCUAUCCUAGAA | [hsa-miR-331-3p](http://mirdb.org/cgi-bin/search.cgi?searchType=miRNA&searchBox=hsa-miR-331-3p) |
| 20538158 | 6.114645 | 0.003061 | 0.0377502 | U29 | chr11:62621376-62621440 (-) | 65 | TTTCTATGATGAATCAAACTAGCTCACTATGACCGACAGTGAAAATACATGAACACCTGAGAAAC | --- |
| 20538162 | 6.083388 | 2.595E-07 | 4.867E-05 | U32A | chr19:49993225-49993301 (+) | 77 | GTCAGTGATGAGCAACATTCACCATCTTTCGTTTGAGTCTCACGGCCATGAGATCAACCCCATGCACCGCTCTGAGA | --- |
| 20518892 | 5.935765 | 0.0218799 | 0.1413856 | hsa-miR-4497 | chr12:110271155-110271171 (+) | 17 | CUCCGGGACGGCUGGGC | [hsa-miR-4497](http://mirdb.org/cgi-bin/search.cgi?searchType=miRNA&searchBox=hsa-miR-4497) |
| 20526178 | 5.884297 | 0.0011094 | 0.0191223 | hsa-miR-7110-5p | chr3:122880646-122880666 (+) | 21 | UGGGGGUGUGGGGAGAGAGAG | [hsa-miR-7110-5p](http://mirdb.org/cgi-bin/search.cgi?searchType=miRNA&searchBox=hsa-miR-7110-5p) |
| 20503809 | 5.834044 | 0.0488573 | 0.2349277 | hsa-miR-497-5p | chr17:6921298-6921318 (-) | 21 | CAGCAGCACACUGUGGUUUGU | [hsa-miR-497-5p](http://mirdb.org/cgi-bin/search.cgi?searchType=miRNA&searchBox=hsa-miR-497-5p) |
| 20500171 | 5.809069 | 0.0151388 | 0.1125001 | hsa-miR-92a-3p | chr13:92003615-92003636 (+) /// chrX:133303574-133303595 (-) | 22 | UAUUGCACUUGUCCCGGCCUGU | [hsa-miR-92a-3p](http://mirdb.org/cgi-bin/search.cgi?searchType=miRNA&searchBox=hsa-miR-92a-3p) |
| 20515627 | 5.742503 | 0.0001155 | 0.0036991 | hsa-miR-320e | chr19:47212551-47212568 (-) | 18 | AAAGCUGGGUUGAGAAGG | [hsa-miR-320e](http://mirdb.org/cgi-bin/search.cgi?searchType=miRNA&searchBox=hsa-miR-320e) |
| 20538177 | 5.670881 | 0.0031494 | 0.038328 | U42A | chr17:27050452-27050509 (+) | 58 | AATGATGGAAAAATCATTATTGGAAAAGAATGACATGAACAAAGGAACCACTGAAGTG | --- |
| 20519433 | 5.299656 | 0.0059541 | 0.0610339 | hsa-miR-4651 | chr7:75544524-75544543 (+) | 20 | CGGGGUGGGUGAGGUCGGGC | [hsa-miR-4651](http://mirdb.org/cgi-bin/search.cgi?searchType=miRNA&searchBox=hsa-miR-4651) |
| 20505791 | 5.299143 | 0.0012717 | 0.020982 | hsa-miR-877-5p | chr6:30552109-30552128 (+) | 20 | GUAGAGGAGAUGGCGCAGGG | [hsa-miR-877-5p](http://mirdb.org/cgi-bin/search.cgi?searchType=miRNA&searchBox=hsa-miR-877-5p) |
| 20500141 | 5.206913 | 0.0080899 | 0.0762115 | hsa-miR-21-5p | chr17:57918634-57918655 (+) | 22 | UAGCUUAUCAGACUGAUGUUGA | [hsa-miR-21-5p](http://mirdb.org/cgi-bin/search.cgi?searchType=miRNA&searchBox=hsa-miR-21-5p) |
| 20500715 | 5.178285 | 0.0001882 | 0.0052443 | hsa-let-7i-5p | chr12:62997471-62997492 (+) | 22 | UGAGGUAGUAGUUUGUGCUGUU | [hsa-let-7i-5p](http://mirdb.org/cgi-bin/search.cgi?searchType=miRNA&searchBox=hsa-let-7i-5p) |
| 20525571 | 5.085122 | 0.0047807 | 0.052605 | hsa-miR-6805-5p | chr19:55899554-55899575 (+) | 22 | UAGGGGGCGGCUUGUGGAGUGU | [hsa-miR-6805-5p](http://mirdb.org/cgi-bin/search.cgi?searchType=miRNA&searchBox=hsa-miR-6805-5p) |
| 20500440 | 4.962399 | 0.0353999 | 0.1937218 | hsa-miR-10b-5p | chr2:177015057-177015079 (+) | 23 | UACCCUGUAGAACCGAAUUUGUG | [hsa-miR-10b-5p](http://mirdb.org/cgi-bin/search.cgi?searchType=miRNA&searchBox=hsa-miR-10b-5p) |
| 20520351 | 4.948696 | 0.0213725 | 0.1394793 | hsa-miR-1273g-3p | chr1:53406042-53406062 (+) | 21 | ACCACUGCACUCCAGCCUGAG | [hsa-miR-1273g-3p](http://mirdb.org/cgi-bin/search.cgi?searchType=miRNA&searchBox=hsa-miR-1273g-3p) |
| 20501176 | 4.837135 | 0.0386839 | 0.2038416 | hsa-miR-99b-5p | chr19:52195871-52195892 (+) | 22 | CACCCGUAGAACCGACCUUGCG | [hsa-miR-99b-5p](http://mirdb.org/cgi-bin/search.cgi?searchType=miRNA&searchBox=hsa-miR-99b-5p) |
| 20500486 | 4.803760 | 0.0142792 | 0.109166 | hsa-miR-222-3p | chrX:45606442-45606462 (-) | 21 | AGCUACAUCUGGCUACUGGGU | [hsa-miR-222-3p](http://mirdb.org/cgi-bin/search.cgi?searchType=miRNA&searchBox=hsa-miR-222-3p) |
| 20534237 | 4.454688 | 0.0025193 | 0.0338534 | HBII-289 | chr2:101889398-101889511 (-) | 114 | ACTGAGGAATGATGACAAGAAAAGGCCGAATTGCAGTGTCTCCATCAGCAGTTTGCTCTCCATGGGCACACGATGACAAAATATCCTGAAGCGAACCACTAGTCTGACCTCAGT | --- |
| 20500399 | 4.338168 | 0.0124081 | 0.0998917 | hsa-miR-199a-5p | chr1:172113732-172113754 (-) /// chr19:10928145-10928167 (-) | 23 | CCCAGUGUUCAGACUACCUGUUC | [hsa-miR-199a-5p](http://mirdb.org/cgi-bin/search.cgi?searchType=miRNA&searchBox=hsa-miR-199a-5p) |
| 20534236 | 4.275930 | 0.0045968 | 0.0510613 | HBII-276 | chr8:67834709-67834784 (-) | 76 | ACAATGATGACTTAAATTACTTTTTGCCGTTTACCCAGCTGAGGTTGTCTTTGAAGAAATAATTTTAAGACTGAGA | --- |
| 20500137 | 4.246108 | 0.0357257 | 0.1941916 | hsa-miR-19b-3p | chr13:92003499-92003521 (+) /// chrX:133303713-133303735 (-) | 23 | UGUGCAAAUCCAUGCAAAACUGA | [hsa-miR-19b-3p](http://mirdb.org/cgi-bin/search.cgi?searchType=miRNA&searchBox=hsa-miR-19b-3p) |
| 20534233 | 4.184315 | 0.00047 | 0.0103697 | HBII-239 | chr16:71792305-71792390 (-) | 86 | TGTGTGTTGGAGGATGAAAGTACGGAGTGATCCATCGGCTAAGTGTCTTGTCACAATGCTGACACTCAAACTGCTGACAGCACACG | --- |
| 20501280 | 4.064035 | 0.011656 | 0.0963867 | hsa-miR-342-3p | chr14:100576052-100576074 (+) | 23 | UCUCACACAGAAAUCGCACCCGU | [hsa-miR-342-3p](http://mirdb.org/cgi-bin/search.cgi?searchType=miRNA&searchBox=hsa-miR-342-3p) |
| 20500162 | 4.032333 | 0.0392279 | 0.2053506 | hsa-miR-30a-5p | chr6:72113298-72113319 (-) | 22 | UGUAAACAUCCUCGACUGGAAG | [hsa-miR-30a-5p](http://mirdb.org/cgi-bin/search.cgi?searchType=miRNA&searchBox=hsa-miR-30a-5p) |
| 20500761 | 4.018901 | 0.0168648 | 0.1196105 | hsa-miR-191-5p | chr3:49058105-49058127 (-) | 23 | CAACGGAAUCCCAAAAGCAGCUG | [hsa-miR-191-5p](http://mirdb.org/cgi-bin/search.cgi?searchType=miRNA&searchBox=hsa-miR-191-5p) |
| 20500115 | 3.826838 | 5.934E-06 | 0.0004558 | hsa-let-7b-5p | chr22:46509571-46509592 (+) | 22 | UGAGGUAGUAGGUUGUGUGGUU | [hsa-let-7b-5p](http://mirdb.org/cgi-bin/search.cgi?searchType=miRNA&searchBox=hsa-let-7b-5p) |
| 20500735 | 3.801366 | 0.0015934 | 0.0247184 | hsa-miR-130a-3p | chr11:57408725-57408746 (+) | 22 | CAGUGCAAUGUUAAAAGGGCAU | [hsa-miR-130a-3p](http://mirdb.org/cgi-bin/search.cgi?searchType=miRNA&searchBox=hsa-miR-130a-3p) |
| 20500123 | 3.664457 | 0.0001788 | 0.0051256 | hsa-let-7f-5p | chr9:96938635-96938656 (+) /// chrX:53584207-53584228 (-) | 22 | UGAGGUAGUAGAUUGUAUAGUU | [hsa-let-7f-5p](http://mirdb.org/cgi-bin/search.cgi?searchType=miRNA&searchBox=hsa-let-7f-5p) |
| 20518878 | 3.505906 | 0.0149403 | 0.111592 | hsa-miR-4484 | chr10:127508372-127508391 (+) | 20 | AAAAGGCGGGAGAAGCCCCA | [hsa-miR-4484](http://mirdb.org/cgi-bin/search.cgi?searchType=miRNA&searchBox=hsa-miR-4484) |
| 20500117 | 3.485834 | 2.721E-06 | 0.0002669 | hsa-let-7c-5p | chr21:17912158-17912179 (+) | 22 | UGAGGUAGUAGGUUGUAUGGUU | [hsa-let-7c-5p](http://mirdb.org/cgi-bin/search.cgi?searchType=miRNA&searchBox=hsa-let-7c-5p) |
| 20519507 | 3.484158 | 0.0152271 | 0.1128926 | hsa-miR-4695-5p | chr1:19209744-19209765 (-) | 22 | CAGGAGGCAGUGGGCGAGCAGG | [hsa-miR-4695-5p](http://mirdb.org/cgi-bin/search.cgi?searchType=miRNA&searchBox=hsa-miR-4695-5p) |
| 20500183 | 3.290784 | 0.0014326 | 0.0226096 | hsa-miR-100-5p | chr11:122022983-122023004 (-) | 22 | AACCCGUAGAUCCGAACUUGUG | [hsa-miR-100-5p](http://mirdb.org/cgi-bin/search.cgi?searchType=miRNA&searchBox=hsa-miR-100-5p) |
| 20500400 | 3.094541 | 0.0041959 | 0.0475184 | hsa-miR-199a-3p | chr1:172113694-172113715 (-) /// chr19:10928105-10928126 (-) | 22 | ACAGUAGUCUGCACAUUGGUUA | [hsa-miR-199a-3p](http://mirdb.org/cgi-bin/search.cgi?searchType=miRNA&searchBox=hsa-miR-199a-3p) |
| 20500458 | 3.094541 | 0.0041959 | 0.0475184 | hsa-miR-199b-3p | chr9:131007024-131007045 (-) | 22 | ACAGUAGUCUGCACAUUGGUUA | [hsa-miR-199b-3p](http://mirdb.org/cgi-bin/search.cgi?searchType=miRNA&searchBox=hsa-miR-199b-3p) |
| 20526172 | -3.009847 | 0.0426293 | 0.2181417 | hsa-miR-7107-5p | chr12:121882129-121882150 (-) | 22 | UCGGCCUGGGGAGGAGGAAGGG | [hsa-miR-7107-5p](http://mirdb.org/cgi-bin/search.cgi?searchType=miRNA&searchBox=hsa-miR-7107-5p) |
| 20533275 | -3.059801 | 0.0166245 | 0.1187672 | ENSG00000238414 | chr10:27157094-27157193 (+) | 100 | ATCCTTTCATAGTTCATAAGCATGATTGGGTTTTCACACTATGTGTAAGATGTGCCTCCATCAAACCTTGTTAGGATGTTGGCATATCACCCATCTGATG | --- |
| 20517729 | -3.254807 | 0.0015892 | 0.0247143 | hsa-miR-4270 | chr3:15537786-15537805 (-) | 20 | UCAGGGAGUCAGGGGAGGGC | [hsa-miR-4270](http://mirdb.org/cgi-bin/search.cgi?searchType=miRNA&searchBox=hsa-miR-4270) |
| 20519589 | -3.258352 | 5.15E-05 | 0.0020915 | hsa-miR-4739 | chr17:77681025-77681049 (-) | 25 | AAGGGAGGAGGAGCGGAGGGGCCCU | [hsa-miR-4739](http://mirdb.org/cgi-bin/search.cgi?searchType=miRNA&searchBox=hsa-miR-4739) |
| 20525511 | -3.433196 | 6.5E-05 | 0.0024629 | hsa-miR-6775-5p | chr16:87868237-87868261 (-) | 25 | UCGGGGCAUGGGGGAGGGAGGCUGG | [hsa-miR-6775-5p](http://mirdb.org/cgi-bin/search.cgi?searchType=miRNA&searchBox=hsa-miR-6775-5p) |
| 20519493 | -3.704101 | 1.914E-05 | 0.0010092 | hsa-miR-4687-3p | chr11:3877343-3877363 (+) | 21 | UGGCUGUUGGAGGGGGCAGGC | [hsa-miR-4687-3p](http://mirdb.org/cgi-bin/search.cgi?searchType=miRNA&searchBox=hsa-miR-4687-3p) |
| 20525585 | -4.266617 | 0.0115817 | 0.0959028 | hsa-miR-6812-5p | chr20:44054155-44054179 (+) | 25 | AUGGGGUGAGAUGGGGAGGAGCAGC | [hsa-miR-6812-5p](http://mirdb.org/cgi-bin/search.cgi?searchType=miRNA&searchBox=hsa-miR-6812-5p) |
| 20518931 | -4.290618 | 0.0029445 | 0.0372764 | hsa-miR-4530 | chr19:39900269-39900286 (-) | 18 | CCCAGCAGGACGGGAGCG | [hsa-miR-4530](http://mirdb.org/cgi-bin/search.cgi?searchType=miRNA&searchBox=hsa-miR-4530) |
| 20536308 | -4.733271 | 0.0020088 | 0.0287175 | hsa-mir-320e | chr19:47212550-47212602 (-) | 53 | GCCUUCUCUUCCCAGUUCUUCCUGGAGUCGGGGAAAAGCUGGGUUGAGAAGGU | [hsa-mir-320e](http://mirdb.org/cgi-bin/search.cgi?searchType=miRNA&searchBox=hsa-mir-320e) |
| 20515550 | -4.819501 | 7.063E-05 | 0.0025586 | hsa-miR-3141 | chr5:153975605-153975623 (-) | 19 | GAGGGCGGGUGGAGGAGGA | [hsa-miR-3141](http://mirdb.org/cgi-bin/search.cgi?searchType=miRNA&searchBox=hsa-miR-3141) |
| 20533693 | -5.273433 | 0.0086279 | 0.0784755 | ENSG00000239055 | chr9:131511637-131511740 (+) | 104 | ATCCTTTCGTAGTTTATAAGAGTGATGATTAGGTCTTCATGCTCATGTGTGAAATGTGCCTCCCTCAAACCATGTTAGGACGTTGGCATATTGCCCATCTGAAA | --- |
| 20529139 | -5.365566 | 0.000116 | 0.0036991 | hsa-miR-7847-3p | chr11:1901335-1901355 (+) | 21 | CGUGGAGGACGAGGAGGAGGC | [hsa-miR-7847-3p](http://mirdb.org/cgi-bin/search.cgi?searchType=miRNA&searchBox=hsa-miR-7847-3p) |
| 20518839 | -5.983361 | 1.208E-05 | 0.000722 | hsa-miR-4459 | chr5:53371390-53371411 (-) | 22 | CCAGGAGGCGGAGGAGGUGGAG | [hsa-miR-4459](http://mirdb.org/cgi-bin/search.cgi?searchType=miRNA&searchBox=hsa-miR-4459) |
| 20517680 | -6.087952 | 6.796E-05 | 0.0025193 | hsa-miR-4298 | chr11:1880735-1880756 (-) | 22 | CUGGGACAGGAGGAGGAGGCAG | [hsa-miR-4298](http://mirdb.org/cgi-bin/search.cgi?searchType=miRNA&searchBox=hsa-miR-4298) |
| 20537464 | -6.712557 | 6.67E-05 | 0.0024993 | hsa-mir-6722 | chr9:139641345-139641422 (-) | 78 | GGCCUCAGGCAGGCGCACCCGACCACAUGCAUGGCUGGUGGCGGCGUGCAGGGGUCGGGUGGGCCAGGCUGUGGGGCG | [hsa-mir-6722](http://mirdb.org/cgi-bin/search.cgi?searchType=miRNA&searchBox=hsa-mir-6722) |
| 20526885 | -7.316623 | 0.0004752 | 0.0104128 | hsa-miR-7162-3p | chr10:30657526-30657544 (-) | 19 | UCUGAGGUGGAACAGCAGC | [hsa-miR-7162-3p](http://mirdb.org/cgi-bin/search.cgi?searchType=miRNA&searchBox=hsa-miR-7162-3p) |
| 20529785 | -8.034027 | 0.0004485 | 0.0099641 | hsa-miR-8075 | chr13:113917280-113917303 (+) | 24 | UGCUGAUGGCAGAUGUCGGGUCUG | [hsa-miR-8075](http://mirdb.org/cgi-bin/search.cgi?searchType=miRNA&searchBox=hsa-miR-8075) |
| 20525426 | -8.134957 | 0.0001405 | 0.0042663 | hsa-miR-6732-5p | chr1:37945836-37945855 (+) | 20 | UAGGGGGUGGCAGGCUGGCC | [hsa-miR-6732-5p](http://mirdb.org/cgi-bin/search.cgi?searchType=miRNA&searchBox=hsa-miR-6732-5p) |
| 20533260 | -10.605058 | 0.0002706 | 0.0069841 | ENSG00000238388 | chr11:100805005-100805109 (+) | 105 | ATCCTTTTGTAGTTCATGGGCATGATGTTTGGGTGTTCACGCATGTGTGTGAGACATGCCACCCTCTGAACCTTGTTACAATCTCAGCATATTACCCGTCTAACC | --- |
| 20533259 | -11.789890 | 0.0002581 | 0.006751 | ENSG00000238388 | chr11:100805005-100805109 (+) | 105 | ATCCTTTTGTAGTTCATGGGCATGATGTTTGGGTGTTCACGCATGTGTGTGAGACATGCCACCCTCTGAACCTTGTTACAATCTCAGCATATTACCCGTCTAACC | --- |
| 20533757 | -12.874522 | 0.0001604 | 0.0047592 | ENSG00000239154 | chr11:101929036-101929139 (-) | 104 | ATCCTTTTGTAGTTTATGAGCATGATGACTGGGTTTTCACAGGTATGTGTGAGATGTGCCATCCTCGAACCTTGTTATGATGTCGGCATATTGTCAGTCTGACA | --- |
