# Supplementary material for: MicroRNAs as potential indicators of the development and progression of uterine leiomyoma
Source: PLoS One. 2022 May 31;17(5):e0268793. doi: 10.1371/journal.pone.0268793 (PMC9154092; doi:10.1371/journal.pone.0268793)
Supplement: S3 Table — (DOCX) [file pone.0268793.s007.docx]

**S3 Table. Correlation between miR expressions with size of leiomyoma (n=16).**

| **Correlation** | **miR-181a-5p** | **miR-127-3p** | **miR-28-3p** | **miR-30b-5p** | **miR-let7c5-p** |
| --- | --- | --- | --- | --- | --- |
| r | 0.516 | 0.516 | 0.499 | 0.492 | 0.478 |
| *P* | 0.024 | 0.024 | 0.030 | 0.032 | 0.038 |

r, correlation coefficient by Pearson’s correlation analysis
